# Changes in the Microbiota of the Scale Insect (*Diaspis echinocacti*, Bouché, 1833) in *Opuntia stricta* Cladodes: Taxonomic and Metagenomic Analysis as a Function of Infestation Levels

**DOI:** 10.3390/biology14091233

**Published:** 2025-09-10

**Authors:** Mikaelly Batista da Silva, Ana Beatriz Medeiros, Antonia Isabelly Monteiro dos Anjos, João Vitor Ferreira Cavalcante, Bianca Cristiane Ferreira Santiago, Shênia Santos Monteiro, Antonio Carlos Vital, Rodrigo Juliani Siqueira Dalmolin, Hugo M. Lisboa, Matheus Augusto de Bittencourt Pasquali

**Affiliations:** 1 Departamento de Engenharia e Gestão de Recursos Naturais, Universidade Federal de Campina Grande, Campina Grande 58401-490, Brazil; 2 Departamento de Engenharia de Alimentos, Universidade Federal de Campina Grande, Campina Grande 58401-490, Brazil; 3 Ambiente Multidisciplinar de Bioinformática—IMD, Universidade Federal do Rio Grande do Norte, Natal 59078-400, Brazil; 4 Departamento de Bioquímica—CB, Universidade Federal do Rio Grande do Norte, Natal 59078-970, Brazilrodrigo.dalmolin@imd.ufrn.br (R.J.S.D.)

**Keywords:** *Diaspis echinocacti*, 16S rRNA, endosymbionts, insect microbiota, *Candidatus Uzinura*

## Abstract

Drought-tolerant cactus *Opuntia stricta* sustains livestock in Brazil’s semi-arid Northeast but suffers yield losses from the armored scale insect *Diaspis echinocacti*. Symbiotic bacteria are thought to underpin scale fitness; however, their response to pest pressure remains unexplored. We characterized the bacterial communities of *D. echinocacti* collected from cladodes displaying low, intermediate, and high infestation (*n* = 3 replicates per level) using 16S-rRNA amplicon sequencing, processed with nf-core/ampliseq. Shannon diversity declined from low to high density, and Bray–Curtis ordination suggested compositional shifts, although group differences were not significant (Kruskal–Wallis and PERMANOVA, *p* > 0.05). The obligate endosymbiont “*Candidatus Uzinura*” dominated all samples (>85% relative abundance) irrespective of density, indicating a resilient core microbiome. PICRUSt2 predicted a contraction of metabolic breadth at higher infestations, with convergence on energy- and amino acid biosynthesis pathways. Taken together, increasing pest density was associated with modest loss of diversity and functional streamlining, rather than wholesale turnover. These baseline data can guide future work on microbiome-based strategies to complement existing scale-insect control in dryland cactus systems.

## Simple Summary

Insect pests can cause substantial damage to crops, and their associated microbiota may play a pivotal role in host physiology and plant interactions. In this study, we investigated the bacterial communities associated with the armored scale insect (*Diaspis echinocacti*) infesting *Opuntia stricta* in Brazil. By comparing insects collected from cladodes under low, medium, and high infestation levels, we assessed microbiota composition using *16S rRNA* gene-based analyses. Our results revealed a marked dominance of *Candidatus Uzinura diaspidicola* across all infestation levels, suggesting a critical symbiotic role in host survival. A potential decline in bacterial diversity with increasing infestation was observed, although this trend was not statistically robust given the limited sample size. These findings provide preliminary insights into the microbiota of scale insects and their possible involvement in host–plant interactions. Further studies employing larger sample sizes and integrative genomic approaches are required to validate these patterns and may ultimately inform microbiome-based strategies for sustainable cactus protection and food security.

## 1. Introduction

*Opuntia stricta*, a cactus species native to Mexico, is highly adapted to semi-arid and arid regions. These regions cover approximately 37% of the Earth’s surface and face significant challenges for agriculture and livestock because of high temperatures, irregular rainfall, and prolonged droughts. Known as fodder cactus, *O. stricta* serves as a vital feed resource for livestock during dry seasons, playing a fundamental role in agricultural sustainability, particularly in Brazil’s semi-arid Northeast [1].

Beyond its nutritional value, the fodder cactus holds ecological, economic, and cultural significance by promoting food security, income generation, and sustainable production alternatives [2,3,4]. However, its cultivation is increasingly threatened by infestations of the armored scale insect *Diaspis echinocacti* (Hemiptera: Diaspididae), which attacks the cladodes and impairs plant growth and productivity. This pest is particularly harmful because of its high reproductive rate, rapid spread under arid climatic conditions, and the waxy carapace that affords females considerable resistance to insecticides [4,5].

Despite the agronomic importance of this pathosystem, no published study has yet examined how the microbiota of *D. echinocacti* changes along a gradient of infestation intensity on *O. stricta*, nor how such changes might modulate plant–insect interactions. This knowledge gap hampers the design of microbiome-informed control strategies that could complement classical biological or chemical measures.

The extensive and often indiscriminate use of chemical insecticides has already selected for resistant *D. echinocacti* populations and raised concerns about toxic residues in animal products derived from livestock fed on infested cacti [6]. Understanding the mechanisms underlying pest adaptation and survival, particularly the role of microbial symbionts, is therefore crucial. Similar to many insects, scale insects harbor both endo- and ectosymbiotic microorganisms that provide essential nutrients, modulate immunity, and influence host development [7,8,9].

Armored scale insects, including *D. echinocacti*, rely on obligate bacterial endosymbionts housed within specialized bacteriocytes that form the bacteriome, a dedicated organ for hosting intracellular symbionts. The primary endosymbiont, *Candidatus Uzinura diaspidicola*, provides essential amino acids and other nutrients critical for host survival on nutrient-poor plant sap, rather than residing in the gut or shifting with feeding behavior [10]. This symbiotic relationship, first characterized by Gruwell et al. [11], is a hallmark of Diaspididae, with *Uzinura* showing high genomic integration with its host, including gene loss and compensatory metabolic pathways. Sabree et al. [12] further confirmed *Uzinura’s* role in nutrient provisioning, particularly in metabolizing plant-derived compounds. Additional studies, such as Szklarzewicz et al. [13] and Bosch et al. [14], highlight the structural and functional stability of bacteriome-hosted endosymbionts in scale insects, though secondary symbionts may modulate immunity or detoxify plant defenses. Understanding these microbial associations is critical for elucidating pest fitness and developing targeted control strategies.

High-throughput sequencing of the *16S rRNA* gene has revolutionized the study of insect-associated microbiota by providing an efficient and cost-effective snapshot of community structure [15]. Nonetheless, analytical choices strongly influence downstream ecological inferences; benchmark comparisons—such as the multi-pipeline assessment by Straub et al. [16]—have shown that different amplicon workflows can bias estimates of diversity and taxon prevalence. In the present work, we therefore applied the nf-core/ampliseq pipeline, which performed favorably in that benchmark, while remaining aware of its limitations.

Although interest in pest-associated microbiota is growing, most recent studies have focused on other Hemipterans (e.g., *Puto barberi*; [17]) or the efficacy of control agents such as detergents and biopesticides against mealybugs (*Drosicha mangiferae*; [18]) without integrating the plant–insect–microbiota triad. Consequently, how infestation intensity shapes *D. echinocacti* symbioses—and how this, in turn, might influence *O. stricta* defense responses—remains poorly characterized.

We therefore hypothesized that microbiota diversity would decline and functional predictions would converge as the intensity of *D. echinocacti* infestation increases on *O. stricta*. Testing this hypothesis will provide baseline information for developing sustainable, microbiome-aware management strategies that reduce chemical dependency and support fodder cactus production in semi-arid regions.

## 2. Materials and Methods

### 2.1. Sample Collection

**Figure 1:**
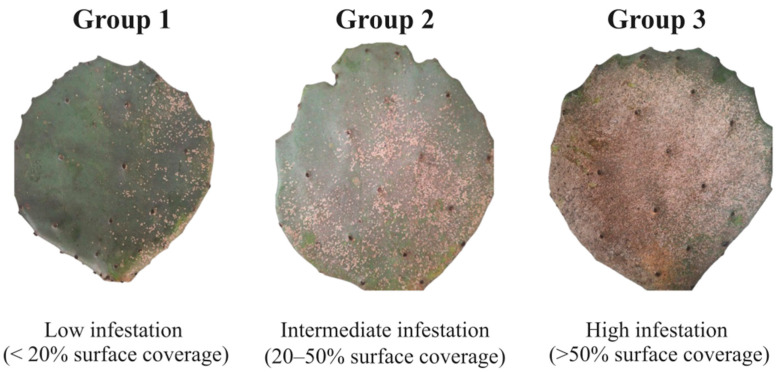
Cladode of *O. stricta* infested by *Diaspis echinocacti*, showing three infestation levels: Group 1 (low infestation, <20% surface coverage), Group 2 (intermediate infestation, 20–50% surface coverage), and Group 3 (high infestation, >50% surface coverage). The magnified detail reveals numerous scale insects adhering to the cladode surface, exhibiting the characteristic circular morphology of the species’ shields. Source: Photo taken by the author.

The insects used in this study were collected from *Opuntia stricta* cladodes from the experimental area of the Instituto Nacional do Semiárido (INSA), located in Campina Grande, Paraíba, Brazil (GPS coordinates: latitude: −7.252411, longitude: −35.946111), in June 2024. The cladodes were classified into three categories based on visual estimation of armored scale insect: Group 1 (low infestation, <20% surface coverage), Group 2 (intermediate infestation, 20–50% surface coverage), and Group 3 (high infestation, >50% surface coverage) (Figure 1). Three biological replicates were collected per infestation level, each consisting of approximately 50 insects pooled from a single cladode to ensure sufficient DNA yield and account for individual variability. Cladodes were selected from different *O. stricta* plants, spaced at least 5 m apart, to ensure spatial independence. No temporal replication was performed due to logistical constraints. Although the selected plants were of similar age, the cladodes varied in age. Cladode age, insect age, and environmental variables such as microhabitat (e.g., sun exposure, soil conditions) and sampling time were not quantitatively controlled in this study. Older cladodes typically exhibit higher levels of *D. echinocacti* infestation [19]. These factors may represent confounding variables that influence the composition of the microbial community. Due to logistical constraints, quantitative assessments and controlled sampling designs were not implemented.

The insects were carefully removed from the *O. stricta* cladodes using forceps, placed in sterile 1.5 mL microcentrifuge tubes, and immediately stored at −20 °C to preserve microbial DNA during transport. The samples were transferred to the laboratory within 24 h, where DNA extraction was performed. Storage at −20 °C was chosen due to logistical constraints that prevented the use of −80 °C facilities. Although immediate extraction or preservation at −80 °C is generally recommended for low-biomass insect samples to reduce potential DNA degradation and changes in microbial community composition, the conditions employed were sufficient to maintain DNA integrity for subsequent analyses.

Surface decontamination of *D. echinocacti* was performed by gently brushing the insects with a sterile soft-bristled brush under a stereomicroscope to remove superficial field debris, such as dust or plant residues, without damaging their delicate structure (approximately 1–2 mm in diameter) or waxy shields. Chemical sterilization methods, such as rinsing with 70% ethanol or 1% sodium hypochlorite, were tested but avoided, as they caused physical damage (e.g., dislodgement of the waxy shield or cuticle degradation) or dragged insects due to surface wetting, compromising sample integrity. For example, ethanol rinsing led to partial shield detachment, potentially exposing internal tissues, while sodium hypochlorite caused visible cuticle erosion. The soft brush method minimized damage while reducing external contaminants, though we acknowledge that it may not eliminate all plant-associated or environmental bacteria, which could influence microbiome profiles. Future studies should explore optimized, non-damaging decontamination protocols for small armored scale insects to further reduce external bacterial interference.

### 2.2. Procedures for 16S Sequencing on the iSeq 100

#### 2.2.1. DNA Extraction and 16S Amplification

DNA was extracted using 3 mg of the insect using the phenol–chloroform method (pH 8.0). Samples were manually macerated with a sterile pestle directly in the microtubes until complete homogenization in the lysis solution, without the use of bead-beating or other mechanical disruption methods. Extraction blanks (negative controls without biological material) were included in all procedures, and no contamination was detected.

To amplify the bacterial 16S, 5 μL of 5X Colorless GoTaq^®^ Flexi Buffer, 0.2 µL of GoTaq^®^ G2 Hot Start Polymerase (Promega, Madison, WI, USA), 1 μL of dNTPs mix (5 mM) (Promega, Madison, WI, USA), 1.6 µL of MgCl_2_, 2 μL of Forward + Reverse primer (5 µM) (synthesized by Integrated DNA Technologies—IDT, Coralville, IA, USA) and 1 µL of extracted DNA were placed in each 0.2 mL microtube for a total volume of 25 µL. The tubes were then placed in the ABI9700 thermal cycler (Applied Biosystems, Foster City, CA, USA) with the following program: 94 °C for 3 min, followed by 40 cycles of 94 °C for 1 min, 53 °C for 1 min, and 72 °C for 2 min. After the 40 cycles, there was a step of 72 °C for 5 min. To check for amplification, the amplicons were subjected to agarose gel electrophoresis (1.5%).

The primer sequences used were as follows:

16S Amplicon PCR Forward Primer = 5′-3′

TCGTCGGCAGCGTCAGATGTGTATAAGAGACAGCCTACGGGNGGCWGCAG

16S Amplicon PCR Reverse Primer = 5′-3′

GTCTCGTGGGCTCGGAGATGTGTATAAGAGACAGGACTACHVGGGTATCTAATCC

#### 2.2.2. Purification and PCR Binding INDEX

The 16S amplicons were purified using AMPure XP magnetic beads (Beckman Coulter, Brea, CA, USA) according to the manufacturer’s recommendations. PCR was then carried out to bind the indexes using the Nextera XT Index kit (Illumina, San Diego, CA, USA). For this, 5 µL of each purified amplicon, 25 µL of 2X PCR BIO Ultra Mix (PCR Biosystems, Wayne, PA, USA), 5 µL of the respective XT index 1 primer (N7xx), and 5 µL of the respective XT index 2 primer (S5xx) were placed in each 0.2 mL microtube for a final volume of 40 µL. The tubes were then placed in the ABI9700 thermal cycler (Applied Biosystems, Foster City, CA, USA) with the following program: 15 °C for 1 min, 95 °C for 3 min, followed by 8 cycles of 95 °C for 30 s, 55 °C for 30 s, and 72 °C for 30 s. After the 8 cycles, there was a step of 72 °C for 5 min. To check for index binding, the amplicons were subjected to agarose gel electrophoresis (1.5%). A second purification was performed after index binding using AMPure XP magnetic beads (Beckman Coulter, Brea, CA, USA) according to the manufacturer’s instructions. Finally, the samples were quantified using the Quantus fluorometer (Promega, Madison, WI, USA) and normalized before being placed on the cartridge for sequencing.

#### 2.2.3. Bioinformatics Analysis

The data, with an average of 118,666 raw reads per replicate, averaging 326,547 for each group, were processed using version 2.10.0 of the nf-core/ampliseq pipeline [16]. For the inference of amplicon sequence variants (ASV), DADA2 [20] was employed, while QIIME2 [21] was used to calculate diversity indices. Following the pipeline’s workflow, reads were first dynamically truncated based on quality (at the first base where the median quality score dropped below 25, while retaining at least 75% of reads) and filtered to discard sequences with a length shorter than 50 bp. Chimeric sequences were then removed using DADA2’s “consensus” method. Taxonomic assignments for the resulting ASVs were made against the SILVA version 138 reference database. Additionally, any ASVs classified as “mitochondria” or “chloroplast” were excluded from the dataset before diversity indices were calculated using QIIME2. This quality control process retained an average of 91.7% of the initial reads.

Taxonomic count tables were obtained by summing the read counts of all ASVs assigned to a given taxon. To assess statistical differences in community composition (beta diversity), a permutational multivariate analysis of variance (PERMANOVA) was performed with 999 permutations. For alpha diversity, group significance was determined using a Kruskal–Wallis test with 2 degrees of freedom, and the resulting pairwise comparisons were adjusted for multiple testing using the False Discovery Rate (FDR) correction. Functional predictions were generated with PICRUSt2 [22] based on *16S rRNA* amplicon sequence variants (ASVs), inferring possible metabolic pathways, such as amino acid biosynthesis and energy metabolism. These predictions represent rough approximations, as accuracy depends on the availability of closely related reference genomes. In communities dominated by poorly characterized endosymbionts, such as *Canidatus Uzinura diaspidicol*, functional inference may be interpreted as hypotheses that require validation through metagenomic or biochemical approaches. All original code has been deposited on GitHub and is publicly available at https://github.com/dalmolingroup/cochineal_16s.

## 3. Results

### 3.1. Analysis of Biological Diversity

**Figure 2:**
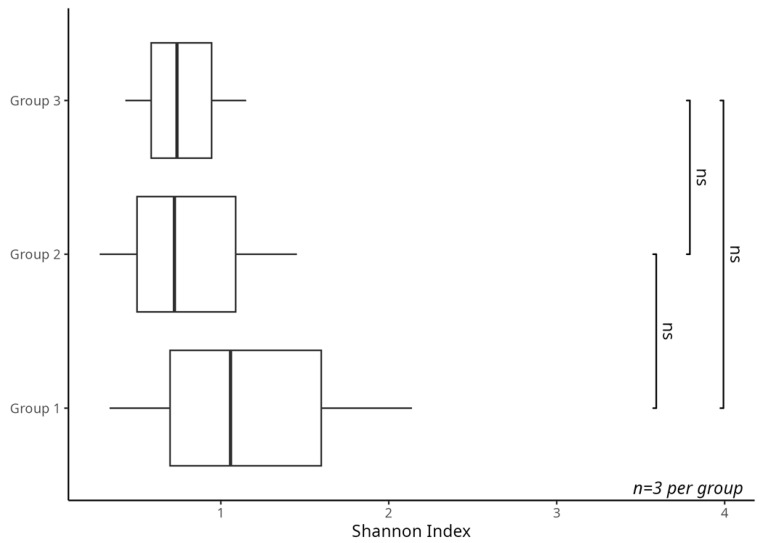
Alpha diversity of the microbiota associated with *D. echinocacti* under different infestation levels, based on the Shannon index. A higher diversity is observed in Group 1 (low infestation), followed by Group 2 (intermediate), and the lowest in Group 3 (high infestation). These results indicate a decline in microbial diversity with increasing infestation, suggesting a potential process of functional specialization. ns: not significant.

The Shannon index indicated a potential trend of higher microbial diversity in the low infestation group (Group 1), with a gradual decrease observed in Groups 2 and 3 (Figure 2). Samples were analyzed in triplicate, accounting for possible developmental variations among individuals. However, the Kruskal–Wallis test showed no statistically significant differences among groups (*p* > 0.05), likely due to the small sample size (*n* = 3 per group), which limits statistical power. These observations are hypothesis-generating and suggest that microbial diversity may decrease with increasing infestation, warranting further investigation with larger sample sizes to confirm biological significance.

In the early stages of infestation, the insect may rely on a broader microbial repertoire to counter host plant defenses. As the infestation progresses, the microbiota becomes increasingly specialized, thereby enhancing the insect’s ability to adapt and become resilient. These microbial shifts likely reflect functional changes aligned with host needs, as reported by Chen et al. [23]. Similarly, Yun et al. [24] demonstrated that the composition of insect gut microbiota is shaped by host development, diet, environment, and phylogeny—factors that not only modulate the presence but also influence microbial function throughout the insect’s life cycle.

Samples were analyzed in triplicate, accounting for possible developmental variations among individuals. Although trends differed between groups, the Kruskal–Wallis test revealed no significant statistical differences (*p* > 0.05), underscoring the need for further integrative studies on insect behavior and microbiota dynamics during infestation.

### 3.2. Taxonomic Composition of the Microbiota Associated with Scale Insects

**Figure 3:**
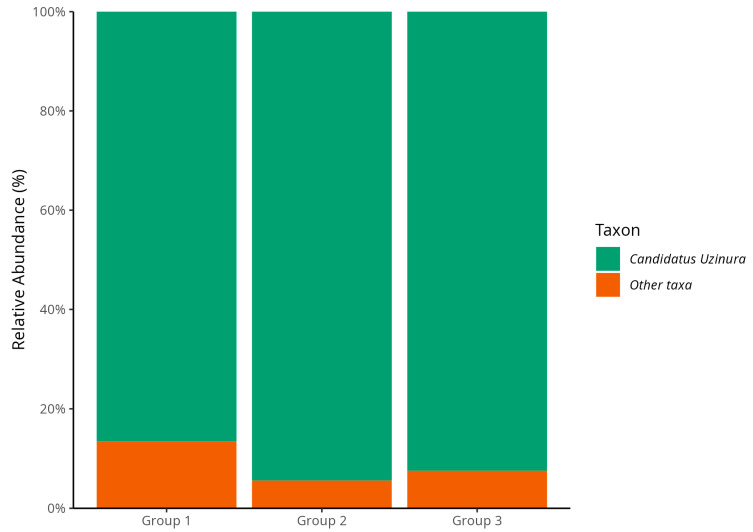
Relative abundance of *C. Uzinura* and other bacterial taxa in the microbiota of *D. echinocacti* across different infestation levels. *C. Uzinura* was the dominant taxon in all groups, especially in Groups 2 and 3 (intermediate and high infestation), where it accounted for nearly the entire microbial community. Group 1 (low infestation) displayed a higher proportion of other taxa, indicating greater microbial diversity at earlier stages of infestation.

The taxonomic composition of the microbiota associated with scale insects was assessed based on both the relative and absolute abundance of taxa, allowing for a comprehensive analysis of differences between infestation groups. The study of relative abundance revealed that *Candidatus Uzinura* was the dominant taxon across all groups, consistent with previous reports identifying it as a common endosymbiont in armored scale insects [12]. The designation “Candidatus” refers to bacteria that, although genetically determined, cannot yet be cultured using conventional laboratory methods. In this study, *C. Uzinura* accounted for 86.4% of the microbial community in Group 1 (low infestation), 94.2% in Group 2 (intermediate infestation), and 92.4% in Group 3 (high infestation), with no statistically significant differences among the groups (Figure 3).

The consistent dominance of *Candidatus Uzinura* across all groups confirms its established role as an obligate endosymbiont in Diaspididae, providing essential nutrients such as amino acids critical for the host’s survival on nutrient-poor plant sap [12]. This aligns with findings by Gruwell et al. [11], who identified *Uzinura* as the primary nutritional symbiont in armored scale insects, and Sabree et al. [12], who demonstrated its metabolic integration with the host. Moreover, the proportion of “other taxa” decreased from Group 1 to Group 2, with a slight increase in Group 3. Although subtle, this trend supports the hypothesis that the microbiome becomes less diverse and increasingly dominated by a single taxon as infestation progresses, characterizing a process of symbiotic specialization.

**Figure 4:**
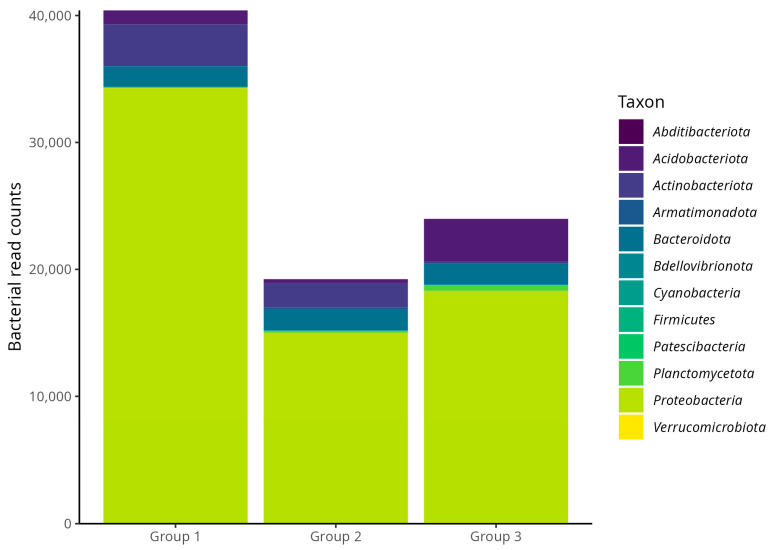
Bacterial read counts at the phylum level associated with the armored scale insect *D. echinocacti* after computational removal of the dominant endosymbiont *Candidatus Uzinura diaspidicola*, shown across different infestation levels (Group 1: low; Group 2: intermediate; Group 3: high). Within this non-symbiont portion of the community, the phylum Proteobacteria was predominant in all groups, especially in Group 1, followed by Acidobacteriota and Bacteroidota. A general decrease in total bacterial read counts was observed from Group 1 to Group 2, with a slight increase in Group 3.

To specifically investigate the composition of the less abundant community members, an analysis was performed on the dataset after computationally removing all reads from the overwhelmingly dominant endosymbiont, *Candidatus Uzinura*. The results of this targeted analysis (Figure 4) show that the phylum Proteobacteria was the most abundant in all groups, particularly in Group 1, followed by Groups 3 and 2. Group 1 showed the highest overall microbial abundance, with notable representation of other phyla such as Actinobacteriota, Acidobacteriota, and Bacteroidota, suggesting a more complex and diverse microbiota at the early stages of infestation. In contrast, Group 2 exhibited a significant reduction in both total abundance and phylum diversity. Group 3 showed partial recovery of total abundance but was dominated by fewer phyla, indicating a more specialized microbial community.

These findings support the hypothesis that microbial diversity and composition are modulated by infestation level. A richer and taxonomically varied microbiota at early infestation stages may aid in insect adaptation and interaction with the host plant. As infestation intensifies, the microbial community becomes simplified, favoring specific symbionts better suited to the host’s stress conditions.

### 3.3. Functional Profile of the Microbiota Associated with Scale Insects

**Figure 5:**
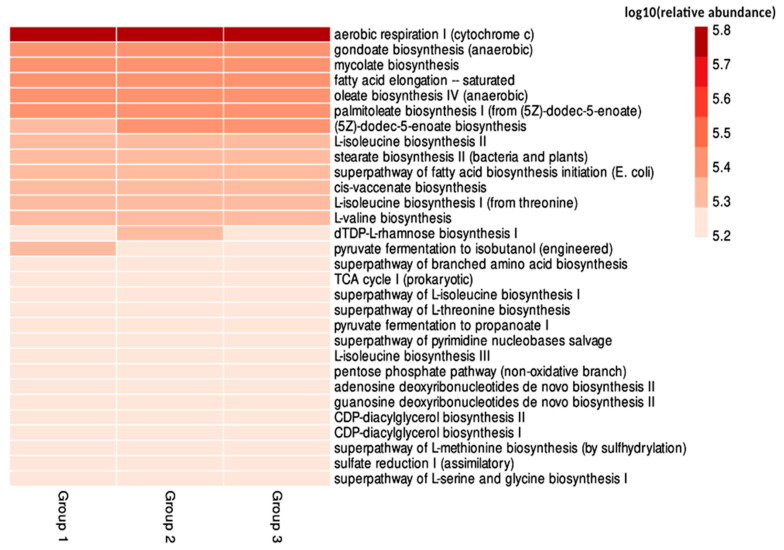
Heatmap showing the 30 most abundant metabolic pathways inferred from the microbiota associated with *D. echinocacti* across three groups of infestation intensity in *O. stricta*. The color gradient represents the log_10_-transformed relative abundance of each metabolic pathway (ranging from 5.2 to 5.8, where darker shades indicate higher abundance). Pathways such as (5Z)-dodec-5-enoate biosynthesis and dTDP-L-rhamnose biosynthesis I show higher abundance in Groups 1 and 2, respectively, reflecting potential functional specialization. Group 1: low infestation; Group 2: moderate infestation; Group 3: high infestation.

The functional potential of the microbiota associated with *D. echinocacti* was inferred using the PICRUSt2 tool, which applies various algorithms to predict gene function based on *16S rRNA* gene sequencing data. The 30 most abundant metabolic pathways across all samples, as expected and referenced in the MetaCyc database [25], are shown in Figure 5.

PICRUSt2-inferred functional profiles suggest a predominance of metabolic pathways related to energy production and biosynthesis, potentially consistent with the genomic capabilities of *Candidatus Uzinura diaspidicola* described by Sabree et al. [12]. Pathways such as aerobic respiration I (cytochrome c), anaerobic gondoate biosynthesis, mycolate biosynthesis, and fatty acid elongation processes showed the highest log_10_-transformed inferred read counts (ranging from 5.2 to 5.8, where higher values indicate greater relative abundance) across all groups, particularly in Group 1 (low infestation). Aerobic respiration I was inferred in all groups, suggesting a conserved metabolic role. In Group 1, the (5Z)-dodec-5-enoate biosynthesis pathway had higher predicted abundance (log_10_ value ~ 5.8), hypothesized to support early-stage colonization through potential microbial signaling or immune enhancement [26,27]. Group 2 exhibited elevated predicted abundance of the dTDP-L-rhamnose biosynthesis I pathway (log_10_ value ~ 5.7), hypothesized to be linked to polysaccharide production for bacterial colonization during intermediate infestation. Group 3 exhibited no significantly elevated predicted pathways, suggesting functional stabilization. These inferences, based on 16S rRNA data, are hypothetical due to the limitations of PICRUSt2, particularly for endosymbionts like *Uzinura* with potentially limited reference genomes, and require validation with metagenomic or biochemical studies.

The predicted functional potential of the microbiota, inferred using PICRUSt2, aligns with the genomic capabilities of *Candidatus Uzinura diaspidicola* described by Sabree et al. [12], which include pathways for synthesizing essential amino acids (e.g., L-isoleucine, L-valine) and fatty acids critical for host nutrition on nutrient-poor plant sap. The predominance of biosynthetic pathways associated with amino acids such as L-isoleucine, L-valine, and L-methionine, especially in Group 1, reflects the metabolic versatility of the microbiota during early infestation stages. This group exhibited a more diverse and abundant functional repertoire compared to Groups 2 and 3, indicating greater metabolic versatility at early stages of infestation. In contrast, the functional diversity in Groups 2 and 3 appeared reduced, reflecting a more specialized or simplified microbial community.

These findings are consistent with the taxonomic results, which revealed greater microbial diversity in Group 1 and dominance of specific taxa in Groups 2 and 3. The predicted reduction in metabolic diversity in samples with higher infestation levels reinforces the hypothesis of a functionally specialized microbiota, possibly co-evolved with the host under more restrictive ecological conditions.

## 4. Discussion

The analysis of alpha diversity in the microbiota associated with *D. echinocacti* revealed a decreasing trend across infestation levels in *O. stricta*. The highest Shannon index was observed in the low-infestation group (Group 1), followed by a gradual decline in Groups 2 and 3. This trend suggests that the microbial community, including the bacteriome-hosted *Candidatus Uzinura* and secondary symbionts, undergoes functional modulation in response to the host insect’s physiological demands, consistent with an adaptive strategy. At early infestation stages, microbial diversity appears to play a crucial adaptive role, supporting the insect in overcoming the plant’s structural and chemical defenses. Symbionts involved in detoxifying secondary metabolites, modulating immunity, and aiding nutrient absorption are essential [24,28]. However, these trends were not statistically significant (*p* > 0.05), likely due to the small sample size (*n* = 3 per group), which limits statistical power.

However, it is important to note that cladode age, which correlates with infestation intensity, may also influence microbial composition, as alder cladodes tend to host more established insect colonies. This confounding factor was not controlled in the present study, and future work should incorporate cladode or insect age as a variable to better clarify its effects. In addition, sample storage at −20 °C for 24 h prior to DNA extraction may have introduced minor alterations in microbial community profiles. Future studies should preferentially employ immediate extraction or −80 °C storage to ensure optimal preservation. Finally, as chemical sterilization methods could damage insect integrity, surface decontamination was performed using a soft brush. While this approach minimizes structural damage, it may have allowed residual plant- or environment-associated bacteria to remain; thus, the development of non-destructive sterilization protocols should be considered in future investigations. Furthermore, functional predictions from PICRUSt2, such as roles in biosynthesis or plant defense neutralization, are coarse approximations, particularly unreliable for endosymbionts like *Uzinura* due to limited reference genomes, and require validation with metagenomics, metatranscriptomics, or targeted assays (e.g., qPCR of functional genes). Future studies should include controls like extraction blanks and plant microbiome analyses to account for external bacteria and employ statistical methods like ANCOM or qPCR-based quantitation to improve compositional and abundance analyses.

This diverse community typically includes generalist bacteria capable of metabolizing plant exudates and neutralizing broad-spectrum defense compounds [29]. As infestation intensifies, a shift toward microbiota specialization is observed, with dominance of symbionts optimized for nutrient extraction and host homeostasis maintenance [6]. This transition aligns with the idea that symbiotic composition adapts to the specific nutritional and defensive context of the host [30]. The stable dominance of *Candidatus Uzinura* in the bacteriome across infestation levels underscores its role as an obligate endosymbiont in Diaspididae, providing essential nutrients and potentially aiding in detoxification of plant defenses, as observed in other scale insects [12,13]. Unlike gut-associated microbiota, which may shift with feeding behavior, *Uzinura’s* intracellular presence in the bacteriome ensures consistent metabolic support regardless of host feeding dynamics. In our study, the reduction in microbial diversity at advanced infestation stages may indicate ecological specialization, which favors functions such as sustained nutrient uptake, immune modulation, and resistance to cumulative defensive compounds, including alkaloids, phenolics, and saponins, produced by cacti [29,31]. These shifts suggest functional adaptations linked to insect development and infestation intensity. Although no statistically significant differences were found (Kruskal–Wallis, *p* > 0.05), the observed trends suggest biologically relevant patterns that may be obscured by individual variability or limitations in sample size. Triplicate analyses were employed to mitigate intraspecific variation; however, future studies with increased statistical power and integrated metagenomics may provide further clarification of these mechanisms.

Microbiota analysis, based on both relative and absolute abundances, revealed changes that aligned with infestation levels, suggesting shifts in ecological and functional community structure. *C. Uzinura* dominated all groups (Figure 3), accounting for 86.4% in Group 1, 94.2% in Group 2, and 92.4% in Group 3. This consistency confirms its role as an obligate symbiont in Diaspididae scale insects [12], likely contributing essential nutrients absent from plant sap [32]. Though primarily nutritional, *Candidatus Uzinura diaspidicola* is hypothesized to potentially contribute to neutralizing plant secondary metabolites in *O. stricta*, based on PICRUSt2-inferred pathways [13,33]. Given PICRUSt2′s limitations for endosymbionts, this role is speculative and requires validation with metagenomics or qPCR-based assays. Its metabolic integration with the insect host is supported by complementary pathways and mutual gene loss and compensation [34,35]. As infestation progresses, changes in plant chemical defenses may require microbiota co-adaptation [36]. A higher proportion of “other taxa” in Group 1, decreasing in later groups, suggests a trend toward specialization. As infestation advances, microbial diversity decreases, favoring *Candidatus Uzinura diaspidicola* in the bacteriome and select secondary symbionts well-adapted to the internal host environment [12,13]. This may reflect ecological filtering, where more efficient mutualist outcompetes less beneficial taxa. The diversity of insect feeding habits and habitats makes it challenging to define a “core” microbiome beyond obligate endosymbionts like *Uzinura*, though specific phyla, including Proteobacteria, Bacteroidetes, Firmicutes, Actinomycota, Spirochaetota, and Verrucomicrobiota, are consistently found across insect taxa [37,38]. Absolute abundance analysis (Figure 4) confirmed that Proteobacteria were the dominant group in all samples, with Group 1 exhibiting the highest representation and most remarkable phylogenetic diversity. This rich, early-stage community likely supports colonization by facilitating key metabolic processes [22]. Other phyla, including Actinobacteriota, Acidobacteriota, and Bacteroidota, were also more prominent in Group 1.

Proteobacteria are involved in degrading organic matter, fermenting sugars, and synthesizing vitamins and aromatic compounds, enhancing nutrient assimilation [39]. Their dominance in various insects, including *Bombyx mori* and *Spodoptera litura*, underscores their central role in the insect microbiome [40,41,42]. Bacteroidetes specialize in breaking down complex polysaccharides, such as cellulose, which facilitates the digestion of lignocellulosic material [43,44]. In *Cyrtotrachelus buqueti*, for example, Bacteroidota contribute glycoside hydrolases that release simple sugars from bamboo-derived polysaccharides. Their presence in *D. echinocacti* suggests a role in digesting complex plant compounds.

Actinobacteria, known for antimicrobial activity, were more abundant in Group 1. These bacteria may support digestion and defense during active feeding stages, as shown in moth larvae [45]. In contrast, Group 2 exhibited a drop in microbial abundance and diversity, likely reflecting physiological stress and disruption of mutualistic associations [22]. Group 3 exhibited partial recovery, but with a dominance of a few phyla, suggesting symbiotic specialization under stress [46]. Overall, the microbiota composition and structure in *D. echinocacti* appear to be modulated by infestation intensity. A diverse early-stage microbiome may promote colonization, while advanced stages involve functional streamlining centered on key symbionts. Endosymbiotic bacteria demonstrate efficient energy metabolism, supported by modular respiratory chains that enable adaptation to host-associated environments [47]. Predictive functional analysis identified the “aerobic respiration I (cytochrome c)” pathway across all groups, suggesting a conserved metabolic core crucial for energy generation. The microbiota also plays roles in immunity, reproduction, and stress resistance [22]. Group-specific pathways further suggest adaptation to host physiology. In Group 1, the exclusive detection of (5Z)-dodec-5-enoate biosynthesis—an unsaturated medium-chain fatty acid—may indicate microbial signaling or immune-enhancing functions [26,27]. In Group 2, the detection of the “dTDP-L-rhamnose I biosynthesis” pathway suggests the production of structural polysaccharides essential for bacterial colonization and immune modulation.

Common detection of amino acid biosynthesis (e.g., L-isoleucine, L-valine, L-threonine), fatty acid elongation, and oleate biosynthesis across groups reflects metabolic functions geared toward cellular homeostasis and membrane stability [48]. In contrast, no exclusive pathways were found in Group 3, suggesting functional consolidation around essential symbiotic functions and reduced redundancy. These findings support the hypothesis of functional succession in the microbiota of *D. echinocacti*, which is shaped by infestation level and the physiological demands of the host insect. Similar functional stability of bacteriome-hosted endosymbionts has been observed in other scale insects, where *Candidatus Uzinura* and related symbionts maintain consistent metabolic roles across developmental stages to support host nutrition and adaptation to plant defenses [12,13,35]. These patterns highlight the conserved role of obligate endosymbionts in Diaspididae, distinct from the more dynamic gut microbiota of other insects. Ultimately, interactions between insects and their endosymbionts drive coevolution, with high genomic integration. In some phloem-feeding insects, key metabolic pathways are partitioned between host and symbiont genomes—a phenomenon known as “collaborative pathways”—which are still largely unexplored in insects with variable diets [49].

## 5. Conclusions

The microbiota of *D. echinocacti* in *O. stricta* shows a potential trend of decreasing diversity with increasing infestation intensity, suggesting possible functional specialization, though these trends were not statistically significant (*p* > 0.05) due to the small sample size (*n* = 3 per group). *Candidatus Uzinura diaspidicola* dominates across all groups, potentially providing essential nutrients and hypothesized to aid in neutralizing plant defenses. These hypothesis-generating findings highlight the potential for microbiome-based pest management strategies in cactus cultivation. Future studies should use larger sample sizes and control for confounders like cladode/insect age and environmental factors (e.g., microhabitat, sampling time) through covariate modeling, stratified sampling by cladode age, or controlled experiments to confirm these trends and elucidate mechanisms of insect–microbiota–plant interactions.

## Data Availability

The raw sequencing data generated in this study have been deposited in the NCBI Sequence Read Archive (SRA) under BioProject accession number PRJNA1309975 (https://www.ncbi.nlm.nih.gov/bioproject/PRJNA1309975).
